# Role and Safety of Tirofiban in Peri-Interventional Antiplatelet Management for Aneurysm Treatment

**DOI:** 10.1007/s00062-024-01480-6

**Published:** 2024-11-28

**Authors:** Rana Garayzade, Ansgar Berlis, Tim Tobias Arndt, Christina Wolfert, Björn Sommer, Gernot Müller, Christoph J. Maurer

**Affiliations:** 1 Department of diagnostic and interventional Neuroradiology, Augsburg University Hospital, Augsburg, Germany; 2 Institute of Mathematics, University of Augsburg, Augsburg, Germany; 3 Department of Neurosurgery, Augsburg University Hospital, Augsburg, Germany; 4 Institute of Mathematics, University of Augsburg. Germany, Augsburg, Germany

**Keywords:** Tirofiban, Emergency aneurysm treatment, Acute Stenting, Thromboembolic complications, Elective aneurysm treatment

## Abstract

**Background:**

Tirofiban is administered for the treatment of aneurysms in cases of thromboembolic complications, as well as in cases of acute stenting or flow-diverter implantation required within the scope of aneurysm treatment. We aimed to investigate the efficacy and safety of tirofiban in this group of patients.

**Methods:**

We conducted a retrospective analysis of all patients undergoing aneurysm treatment and receiving peri-interventional tirofiban administration at our institution between 2009 and 2019.

**Results:**

A total of 105 patients were included, with 61% women and 39% men (mean age = 53 years, IQR: 44–60 years). Sixty-seven patients underwent emergency aneurysm treatment, and thirty-eight were treated electively. Hemorrhagic events occurred in 22% (15/67) of the patients treated acutely, with 7.46% (5/67) exhibiting symptoms. Patients undergoing elective aneurysm treatment experienced no hemorrhagic events (*p* = 0.002). Among the 35 patients who required an external ventricular drain (EVD), 22.86% (8/35) developed EVD-related hemorrhages; however, none were symptomatic (*p* = 0.007). Of the five patients who required a craniotomy, two experienced significant bleeding, and one experienced non-significant craniotomy-related bleeding (*p* = 0.20).

**Conclusion:**

Tirofiban may be safe for use during peri-interventional complications or emergency stenting in aneurysm treatment. However, caution is necessary when craniotomy is required. In elective aneurysm treatments, administering Tirofiban in response to periprocedural complications appears to be safe.

**Supplementary Information:**

The online version of this article (10.1007/s00062-024-01480-6) contains supplementary material, which is available to authorized users.

## Introduction

Endovascular coil embolization is a widely accepted, useful treatment modality for intracranial aneurysms [1, 2]. Stent-assisted coiling (SAC), developed for secure coil packing in large and wide-necked aneurysms, results in lower recurrence, retreatment rates, and hemorrhage rates, and improves outcomes compared to conventional coiling [3, 4]. However, the procedure is linked to higher complication rates and less favorable outcomes when applied to ruptured aneurysms [5]. In addition, flow diversion devices (FDD) appear to be an effective treatment for ruptured, fusiform, blister, and dissecting aneurysms [6].

The placement of a stent or FDD requires dual antiplatelet therapy (DAPT) due to increased thrombogenicity. While less concerning in unruptured cases, managing both, the risks of thrombotic complications and rebleeding in ruptured aneurysms presents a challenge [7]. Most operators are reluctant to use antiplatelet therapy in the setting of acute subarachnoid hemorrhage (aSAH), because of the potential need for an extraventricular drain (EVD), a ventriculostomy, and the high likelihood of future invasive interventions [5].

The optimal antiplatelet therapy is still a matter of debate, but many interventionalist prefer a periprocedural DAPT with acetylsalicylic acid (ASA) and a glycoprotein (GP) IIb/IIIa inhibitor [7]. Alternatively, in the setting of aSAH, prolonged intravenous tirofiban as the sole antiplatelet therapy in the perioperative period for patients undergoing endovascular SAC or FDD placement, has been suggested with a continuous infusion until EVD removal or placement of a permanent ventriculoperitoneal shunt [8].

The aim of this study is to determine whether peri-interventional administration of tirofiban may increase the risk of hemorrhagic events in patients undergoing neuroendovascular treatment for intracranial aneurysms.

## Materials und Methods

### Patient Population

This retrospective single-center study was approved by the ethics committee of Ludwig Maximilian University of Munich (study approval number 21-0218). Due to the retrospective nature of our study formal consent was waived.

We reviewed our institution’s database to identify all patients who underwent endovascular treatment for ruptured and unruptured aneurysms between 2009 and 2019. The study included all patients who received peri-interventional tirofiban. A total of 105 consecutive patients met these inclusion criteria. The treatments were performed or supervised by four operators, whose experience ranged from 7 to 26 years.

### Pre- und Periinterventional Antiplatelet Management by Elective/Unruptured Aneurysms

Until 2015, our institutional guidelines did not recommend premedication for treatments involving coils only. However, for elective cases identified through pre-interventional imaging as potentially or probably requiring stents or FDDs, patients were administered 100 mg of ASA and 75 mg of clopidogrel daily for five days prior to the procedure. Additionally, a multiplate test was conducted on the day of the intervention to evaluate the responder status. For planned treatment with WEB, the pre-interventional regimen was the same as for stent-assisted coiling. Starting in 2015, all patients undergoing elective procedures received DAPT five days prior to the procedure, and confirmation of responder status at the day prior to the intervention. In the case of coil-only interventions, both ASA and clopidogrel were discontinued after the aneurysm therapy.

If the multiplate test indicated non-responder or uncertain responder status, we initiated peri-interventional intravenous administration of tirofiban based on body weight. Patients with responder status received tirofiban in the event of peri-interventional thromboembolic complications.

### Periinterventional Antiplatelet Management for Ruptured Aneurysms

Depending on premedication, tirofiban was administered either due to peri-interventional complications or because of stenting. At the discretion of the operators, some patients additionally received intravenous ASA.

### Periinterventional Anticoagulation Management

Until 2015, we administered periprocedural heparin to achieve a target PTT of 40–60 s. However, at the individual discretion of the operators, some patients did not receive heparin.

### Tirofiban Peri- and Postinterventionally

We employed a high-dose bolus regimen adapted from cardiology practices [9, 10]. Tirofiban was administered intravenously at 25 mcg/kg within 3 min after stenting, followed by a maintenance dose of 0.15 mcg/kg/min for up to 24 h post-procedure, consistent with protocols in major cardiological trials [11, 12]. This regimen continued unless CT scans in the angio suite immediately post-procedure revealed new intracranial hemorrhage (ICH) or aSAH. In cases of severe renal insufficiency (eGFR < 30 ml/min), tirofiban dose was reduced by 50%. In the event of a peri-interventional thromboembolic complication during the procedure, as diagnosed during a control angio run, Tirofiban was administered as a bolus using the same regimen and dosage as before stent placement or, in the case of clopidogrel non-responders, with further infusion after exclusion of relevant post-interventional bleeding using flat detector CT in the angio suite. If an early follow-up CT scan showed significant ICH or severe systemic bleeding leading to clinical deterioration, tirofiban infusion was discontinued.

### Statistics

Descriptive statistics were used to summarize the patient characteristics and outcomes. Continuous variables were described by the median and interquartile range. Categorical variables were expressed as numbers and percentages. Variables were compared with Wilcoxon rank-sum tests for continuous variables and a chi-squared test or Fisher’s exact test for categorical variables.

## Results

### Study Patients and Baseline Characteristics

A total of 105 patients were included in the analysis. The patient cohort consisted of 61% females and 39% males.

Symptomatic aneurysms were present in 67% of the patients, with 61% having aSAH and 3% having symptomatic aneurysms without SAH. Asymptomatic aneurysms were found in 36% of the patients. Most aneurysms were located in the anterior circulation (73%), and the mean aneurysm size was 6.0 mm. Regarding premedication, 53% of patients received no premedication, while 22% were on dual antiplatelet therapy, and 14% were on monotherapy.

Table 1 below provides additional details on the baseline characteristics.

**Table 1:** Baseline characteristics, procedural characteristics

Characteristics	Overall, *N* = 105
*Age Mean in years (IQR)*	53 (44, 60)
*Sex*	
Male	41(39%)
Female	64(61%)
*Symptomatic aneurysms*	
aSAH	64(61%)
Symptomatic w/o SAH	3 (3%)
Asymptomatic	38 (36%)
*Aneurysm location*	
Posterior circulation	23 (22%)
Anterior circulation	77 (73%)
Anterior and posterior circulation	5 (5%)
*Aneurysm size (maximal mm) Mean (IQR)*	6.0 (4.5, 9.0)
*Premedication*	
Dual antiplatelet	14 (13%)
Marcumar/NOAC	2 (2%)
Monotherapy (one antiplatelet)	15 (14%)
No premedication	56 (53%)
Non-responder	18 (17%)

In our cohort, the following materials were used for aneurysm treatment: stent/coils (*n* = 58), coils (*n* = 14), FDD (*n* = 16), coils/FDD (*n* = 7), WEB (*n* = 5), WEB/FDD (*n* = 1), WEB/stent/coils (*n* = 1) and WEB/stent (*n* = 3), *p* = 0.1049. Detailed results are available in Supplementary File Table 1 and 2.14 patients received dual premedication, 15 patients underwent monotherapy (primarily with ASA), 56 patients had no premedication, and 18 patients were non- or partial responders to clopidogrel (*p* = 0.22).

Periinterventionally, 31 patients received only tirofiban, 6 received both tirofiban and ASA, 43 received tirofiban, ASA, and heparin, and 25 received tirofiban and heparin alone, intravenously (*p* = 0.213).

**Fig. 1:**
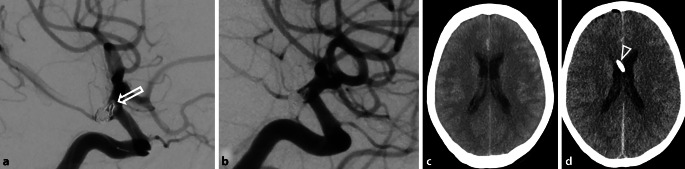
A patient with a ruptured posterior communicating artery (PCOM) aneurysm treated with coiling. **a** After coiling, an appositional thrombus is observed at a coil loop at the aneurysm base (arrow). **b** Resolution of the thrombus after tirofiban administration. **c** CT scan showing bilateral aSAH before coiling and EVD placement. **d** CT scan after EVD placement (*arrowhead*), coiling, and tirofiban administration, without signs of re-hemorrhage

**Fig. 2:**
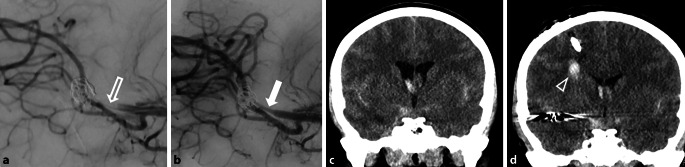
A patient with a ruptured middle cerebral artery (MCA) aneurysm treated with a flow diverter and coils. **a** After the procedure, in-stent thrombosis resulting in occlusion of an MCA branch (*open arrow*). **b** Recanalization of the branch (*closed arrow*) 20 min after initiation of tirofiban administration. **c** CT scan showing bilateral aSAH before EVD placement and endovascular treatment. **d** CT scan after EVD placement, intervention, and tirofiban administration, revealing an asymptomatic catheter tract hemorrhage (*arrowhead*) along the trajectory of a failed EVD placement attempt

Out of 105 patients, 90 (85.71%) did not experience any hemorrhage (Fig. 1), 5 (4.76%) had a symptomatic hemorrhage, and 10 (9.52%) had asymptomatic hemorrhagic complications (Fig. 2).

In the SAH group, more hemorrhagic events were diagnosed compared to the group with symptomatic but unruptured aneurysms (*p* = 0.027), as shown in Table 2.

**Table 2:** No hemorrhage versus any hemorrhage

Characteristics	No hemorrhage *N* = 90	Any hemorrhage *N* = 15	*p*-value
*Age in years (IQR)*	54 (44, 61)	50 (45, 56)	0.30
*Sex*			0.072
Male	32 (78%)	9 (22%)	–
Female	58 (91%)	6 (9.4%)	–
*Symptomatic aneurysms*			0.002*
Yes	52 (78%)	15 (22%)	–
No	38 (100%)	0 (0%)	–
*SAH*			0.027*
Yes	51 (80%)	13 (20%)	–
No	39 (95%)	2 (4.9%)	–
*Aneurysm location*			0.40
Posterior circulation	18 (78%)	5 (22%)	–
Anterior circulation	67 (87%)	10 (13%)	–
Anterior and posterior circulation	5 (100%)	0 (0%)	–
*Aneurysm size (maximal mm)*	6.0 (4.1, 9.0)	6.1 (5.3, 13.0)	0.30
*Modified Fisher Score*			< 0.001*
I	5 (100%)	0 (0%)	–
II	27 (96%)	1 (3.6%)	–
III	2 (33%)	4 (67%)	–
IV	16 (67%)	8 (33%)	–
No SAH	40 (95%)	2 (4.8%)	–
*Craniotomy*			0.001*
Yes	1 (20%)	4 (80%)	–
No	89 (89%)	11 (11%)	–
*Periinterventional ASA or heparin*			0.50
Yes	62 (84%)	12 (16%)	–
No	28 (90%)	3 (9.7%)	–
*Premedication*			0.005*
Yes	47 (96%)	2 (4.1%)	–
No	43 (77%)	13 (23%)	–

*-statistically significant

No significant association was found regarding the rate of symptomatic hemorrhage and patient age or aneurysm location. Nevertheless, a significant association between symptomatic hemorrhage and male sex (*p* = 0.044) as well as aneurysm size (*p* = 0.040) can be inferred (Table 3 and Supplementary Table 3).

**Table 3:** Asymptomatic versus symptomatic hemorrhage

Characteristics	Asymptomatic hemorrhage *N* = 10	Symptomatic hemorrhage *N* = 5	*p*-value
*Age in years (IQR)*	49 (40, 51)	58 (50, 78)	0.10
*Sex*			0.044*
Male	4 (44%)	5 (56%)	–
Female	6 (100%)	0 (0%)	–
*aSAH*			0.10
Yes	10 (77%)	3 (23%)	–
No	0 (0%)	2 (100%)	–
*Aneurysm location*			0.60
Posterior circulation	4 (80%)	1 (20%)	–
Anterior circulation	6 (60%)	4 (40%)	–
*Aneurysm maximum size (in mm)*	5.7 (5.1, 7.5)	17.0 (12.0, 18.0)	0.040*
*Modified Fisher Score*			0.019*
II	0 (0%)	1 (100%)	–
III	4 (100%)	0 (0%)	–
IV	6 (75%)	2 (25%)	–
No	0 (0%)	2 (100%)	–
*Craniotomy*			0.60
Yes	2 (50%)	2 (50%)	–
No	8 (73%)	3 (27%)	–
*Periinterventional ASA or heparin*			> 0.90
Yes	8 (67%)	4 (33%)	–
No	2 (67%)	1 (33%)	–
*Premedication*			0.10
Yes	0 (0%)	2 (100%)	–
No	10 (77%)	3 (23%)	–
*Craniotomy—related hemorrhage*			0.20
Yes	1 (33%)	2 (67%)	–
No	9 (75%)	3 (25%)	–
*EVD-related hemorrhage*			0.007*
Yes	8 (100%)	0 (0%)	–
No	2 (29%)	5 (71%)	–
*EVD timing*			0.13
EVD before intervention	5 (56%)	4 (44%)	–
EVD before and after intervention	5 (100%)	0 (0%)	–
No EVD	0 (0%)	1 (100%)	–

*-statistically significant

### Patients with Symptomatic Hemorrhage

In the first case, a 28 mm symptomatic, fusiform basilar aneurysm was treated using two overlapping flow diverters. Eleven months later, a ventriculo-peritoneal shunt was placed for normal pressure hydrocephalus, and antiplatelet therapy was discontinued four days prior. Postoperative complications resulted in extensive cerebellar and pontine infarcts. Despite thrombectomy and continued tirofiban, the patient progressed to palliative care and passed away.

In the next case, a 17 mm symptomatic ACI aneurysm was treated with two flow diverters. Due to multiple conditions posing a high bleeding risk, only ASA was used for premedication along with peri-interventional tirofiban. Post-procedure, ticagrelor was introduced, but the patient developed a worsening chronic subdural hematoma, requiring evacuation. The clinical decline led to palliative care, and the patient passed away.

Another case involved a large ruptured ophthalmic ICA aneurysm, managed using a flow diverter. The patient received ASA and tirofiban, followed by ticagrelor and ASA. Elevated intracranial pressure from vasospasms necessitated craniectomy and further revisions. Progressive vasospasms and infarctions led to palliative care, and the patient passed away.

A further patient presented with a large ruptured ACOM aneurysm, treated with Y‑stent placement and coil embolization. Peri-procedural tirofiban and ASA were administered, with a planned ticagrelor loading dose. Elevated intracranial pressure and rebleeding were noted, leaving no viable treatment options. The patient passed away.

The last case concerns a patient with aSAH and ICA fusiform dilation considered for FDD treatment, who received ASA and tirofiban. Planned ticagrelor administration was delayed due to worsening symptoms, and a CT revealed an ICH. After conservative management and resuming ticagrelor, the patient improved and achieved a mRS of 3 at discharge.

### Hemorrhage in the Patient Group with EVD and Craniotomy

In our cohort, a total of 35 patients received EVD, with 23 undergoing surgery solely prior to the intervention and 12 receiving it both before and after intervention. Five patients required craniotomy. Among the patients who had EVD, 23% (8/35) experienced EVD-associated hemorrhage. Of note, no patient in this subgroup had symptomatic hemorrhage. Of these, 4 were still on tirofiban at the time of EVD placement, while the remaining patients had already been switched to oral DAPT.

In our cohort, craniotomy was required in 5 patients, 2 for rising ICP due to SAH without progression after intervention, 2 for rising ICP associated with vasospasm and infarction, and 1 for progression of pre-existing cSDH on DAPT. Among the five patients who underwent craniotomy, two exhibited symptomatic bleeding. One of these patients was still receiving tirofiban treatment, while the other was already undergoing DAPT. Additionally, one patient experienced an asymptomatic hemorrhage following craniotomy under tirofiban.

## Discussion

In our study, we utilized tirofiban to manage the risk of thromboembolic complications during aneurysm treatment. Out of 105 patients, 85.71% did not experience any hemorrhagic complications. Symptomatic hemorrhage occurred in 4.76% of patients, while 9.52% had asymptomatic hemorrhages.

Thromboembolic complications during endovascular treatment of cerebral aneurysms are defined as any event involving complete or partial occlusion of arteries at the site of the aneurysm, distal to the vascular territory where the endovascular procedure was performed, or in any other vascular territory [13].

Thromboembolic complications may be caused by clot formation in the guiding catheter, on the coil mashes, or in parent vessels caused by the induced vasospasm or malposition of coils with prolapsed coil loops [13]. Not every aneurysm can be optimally treated through coiling alone. In case of wide-necked aneurysms, pseudoaneurysms, or blister aneurysms, stenting or FDD are required. With their assistance, optimal endovascular treatment of these complex aneurysms is possible [14, 15]. However, thromboembolic complications are more frequent in the application of endovascular stents [16]. In our patient group with ruptured aneurysms, 10 patients received stents or FDD due to dissected (blood-blister like) aneurysm, and 3 patients received them for fusiform aneurysms.

Assessment of antiplatelet activity prior to stent implantation has subsequently reduced the incidence of thrombotic complications. Testing clopidogrel-related platelet inhibition through impedance aggregometry can identify non-responders in up to 28% of patients, revealing a statistical association between clopidogrel non-responders and adverse thromboembolic events [17]. In our cohort, 32 out of 105 patients (30%) had DAPT as premedication. Of these, 18 patients were non- or only partial responsive to clopidogrel. The challenge of ensuring sufficient antiplatelet effect in clopidogrel non-responders has been addressed by periinterventional addition of the second antiplatelet medication, tirofiban. Tirofiban is a glycoprotein IIb/IIIa receptor antagonist. Glycoprotein IIb/IIIa inhibitors were developed on the premise that blockade of the GP IIb/IIIa integrin, which is present on platelets and represents the final pathway of platelet aggregation, is potentially more effective than inhibition of the single activation pathway of platelet aggregation, as is the mechanism of conventional agents such as aspirin, ticlopidine, or clopidogrel [18]. Intravenous tirofiban inhibits platelet aggregation within 5 min, maintains this effect for the duration of the infusion, declines to < 50% four hours after cessation of the infusion and returns to near-baseline levels by eight hours, a finding consistent with the drug’s relatively short terminal elimination half-life [19]. It is important to note that in our cohort, 14 out of 105 patients (13%) were on DAPT with a positive responder status but still experienced thromboembolic complications. In these cases, tirofiban was administered as a third antiplatelet agent. Notably, none of these patients experienced hemorrhagic complications.

In the aSAH group, there were more hemorrhagic events compared to the group with symptomatic but unruptured aneurysms. This is most likely associated with the deranged coagulation due to aSAH and the necessity of EVD drainage and/or craniotomy in this subgroup. So, eight out of ten of all asymptomatic bleedings were EVD-associated, and one was craniotomy-associated. Two out of five symptomatic hemorrhages were craniotomy-associated. It should be emphasized that the large and complex aneurysms in our cohort tended to develop significant bleeding complications. Notably, all five patients with symptomatic hemorrhage were high-risk patients with predominantly symptomatic aneurysms and significant comorbidities. They underwent complex endovascular treatments for cerebral aneurysms, some as a last resort, requiring aggressive antiplatelet and anticoagulant management. Unfortunately, four of the patients had fatal outcomes due to severe complications such as hemorrhagic or ischemic strokes. However, none of the symptomatic hemorrhages were caused by the placement of an EVD.

In recent studies, FDD and stents with special antithrombogenic coating have been increasingly used [20–22]. However, it remains intriguing but unclear whether new antithrombogenic materials could allow tirofiban alone as an antiplatelet agent during acute stenting, potentially reducing the common occurrence of peri-interventional thromboembolic and hemorrhagic complications. This consideration is particularly relevant since tirofiban is typically used in conjunction with aspirin in cases of thromboembolic complications. Future studies are needed to explore the efficacy and safety of these materials in minimizing the need for additional antiplatelet agents.

Our study is limited by several factors. First, it is a retrospective, single-center cohort study with a limited sample size and no control group. Secondly, we focused exclusively on peri-interventional complications and did not include long-term follow-up. These limitations may affect the generalizability and depth of our findings. Despite these limitations, our data suggest a favorable safety profile for Tirofiban in both endovascular treatment of ruptured and unruptured aneurysms.

## Conclusion

Tirofiban may be safely administered for peri-interventional complications or emergency stenting during aneurysm treatment, although caution is necessary when a craniotomy is anticipated. In our cohort, no patients with ruptured aneurysms who received peri-interventional tirofiban experienced symptomatic EVD-associated hemorrhages. Additionally, in elective aneurysm treatments with proper dual antiplatelet premedication, administering tirofiban during periprocedural complications appears to be safe, as demonstrated by the absence of hemorrhagic events.

## Supplementary Information

Supplementary tables 1–3

## Abbreviations

ASA: acetylsalicylic acid
aSAH: aneurysmal subarachnoid hemorrhage
CT: computed tomography
DAPT: dual antiplatelet therapy
EVD: external ventricular drain
FDD: flow diversion device
ICH: intracerebral hemorrhage
WEB: Woven EndoBridge
