# Supplementary material for: Role and Safety of Tirofiban in Peri-Interventional Antiplatelet Management for Aneurysm Treatment
Source: Clin Neuroradiol. 2024 Nov 28;35(2):247–54. doi: 10.1007/s00062-024-01480-6 (PMC12174253; doi:10.1007/s00062-024-01480-6)
Supplement: Supplementary file 1 — Supplementary tables 1–3 [file 62_2024_1480_MOESM1_ESM.docx]

**Role and Safety of Tirofiban in Peri-interventional Antiplatelet Management for Aneurysm Treatment.**

**Content**

**Supplementary table 1.** Premedication and materials used for treatment (with number of patients with thromboembolic complications in parentheses)

**Supplementary table 2.** Periinterventional management (with number of patients with thromboembolic complications in parentheses).

**Supplementary table 3** No symptomatic hemorrhage (no hemorrhage plus asymptomatic hemorrhage) versus symptomatic hemorrhage.

| Premedication | dual antiplatelet(n=14) | monotherapy(n=15) | no premedication(n=56) | NOAK/ Markumar( n=2) | Non-Responder ( n=18) |
| --- | --- | --- | --- | --- | --- |
| FDD (n=16) | 1 (1) | 5(1) | 7(0) | 1(0) | 2(1) |
| stent /coils( n=58) | 9(9) | 5(2) | 30(10) | 1(0) | 13(7) |
| coils( n=14) | 1(1) | 1(1) | 10(10) | 0 | 2(2) |
| WEB/ FDD( n=1) | 1 (1) | 0 | 0 | 0 | 0 |
| WEB/ stent/ coils( n=1) | 1 (1) | 0 | 0 | 0 | 0 |
| WEB( n=5) | 1 (1) | 2(2) | 2(2) | 0 | 0 |
| WEB/ stent( n=3) | 0 | 1(0) | 2(0) | 0 | 0 |
| coils/ FDD( n=7) | 0 | 1(0) | 5(2) | 0 | 1(1) |

**Table 1 :** premedication and materials used for treatment(with number of patients with thromboembolic complications in parentheses)

| Periinterventional Medication | No medication( n=31) | ASA intravenous( n=6) | ASA/ Heparin intravenous (n=43) | Heparin intravenous (n=25) |
| --- | --- | --- | --- | --- |
| FDD(n=16) | 7(1) | 0 | 5(0) | 4(2) |
| stent/ coils( n=58) | 15(8) | 4(0) | 24(11) | 15(9) |
| coils( n=14) | 1(1) | 1(1) | 9(9) | 3(3) |
| WEB/ FDD( n=1) | 1(0) | 0 | 0 | 0 |
| WEB/ stent/ coils( n=1) | 1(1) | 0 | 0 | 0 |
| WEB( n=5) | 2(2) | 0 | 2(2) | 1(1) |
| WEB/ stent( n=3) | 0 | 0 | 2(0) | 1(0) |
| coils/ FDD ( n=7) | 4(2) | 1(0) | 1(0) | 1(1) |

**Table 2 :** periinterventional management (with number of patients with thromboembolic complications in parentheses).

| Characteristics | No symptomatic hemorrhage, N = 100 | Symptomatic hemorrhage  N = 5 | p-value |
| --- | --- | --- | --- |
| **Age Mean in years (IQR)** | 53 (44, 60) | 58 (50, 78) | 0.2 |
| **Sex** | |  | 0.008* |
| male | 36 (88%) | 5 (12%) |  |
| female | 64 (100%) | 0 (0%) |  |
| **Symptomatic aneurysms** | |  | 0.2 |
| yes | 62 (93%) | 5 (7.5%) |  |
| no | 38 (100%) | 0 (0%) |  |
| **aSAH** | |  | >0.9 |
| yes | 61 (95%) | 3 (4.7%) |  |
| no | 39 (95%) | 2 (4.9%) |  |
| **Aneurysma location** | | | >0.9 |
| Posterior circulation | 22 (96%) | 1 (4.3%) |  |
| Anterior circulation | 73 (95%) | 4 (5.2%) |  |
| Anterior and posterior circulation | 5 (100%) | 0 (0%) |  |
| **Aneurysm size ( maximal mm) Mean (IQR)** | 6.0 (4.4, 8.9) | 17.0 (12.0, 18.0) | 0.016* |
| **Modified Fischer Score** | |  | 0.8 |
| I | 5 (100%) | 0 (0%) |  |
| II | 27 (96%) | 1 (3.6%) |  |
| III | 6 (100%) | 0 (0%) |  |
| IV | 22 (92%) | 2 (8.3%) |  |
| no | 40 (95%) | 2 (4.8%) |  |
| **Craniotomy** | | | 0.017* |
| yes | 3 (60%) | 2 (40%) |  |
| no | 97 (97%) | 3 (3.0%) |  |
| **periinterv_ASA_or_heparin** | | | >0.9 |
| yes | 70 (95%) | 4 (5.4%) |  |
| no | 30 (97%) | 1 (3.2%) |  |
| **Premedication** | |  | >0.9 |
| yes | 47 (96%) | 2 (4.1%) |  |
| no | 53 (95%) | 3 (5.4%) |  |

**Table 3.** No symptomatic hemorrhage (no hemorrhage plus asymptomatic) versus symptomatic hemorrhage.
